# Supplementary material for: Metagenomic Analysis Reveals A Gut Microbiota Structure and Function Alteration between Healthy and Diarrheic Juvenile Yaks
Source: Animals (Basel). 2024 Apr 14;14(8):1181. doi: 10.3390/ani14081181 (PMC11047321; doi:10.3390/ani14081181)
Supplement: Supplementary file 1 [file animals-14-01181-s001.zip › animals-2899871-supplementary.pdf]

Metagenomic Analysis Reveals Gut Microbiota Structure and Function Alteration between Healthy and Diarrheic Juvenile Yaks

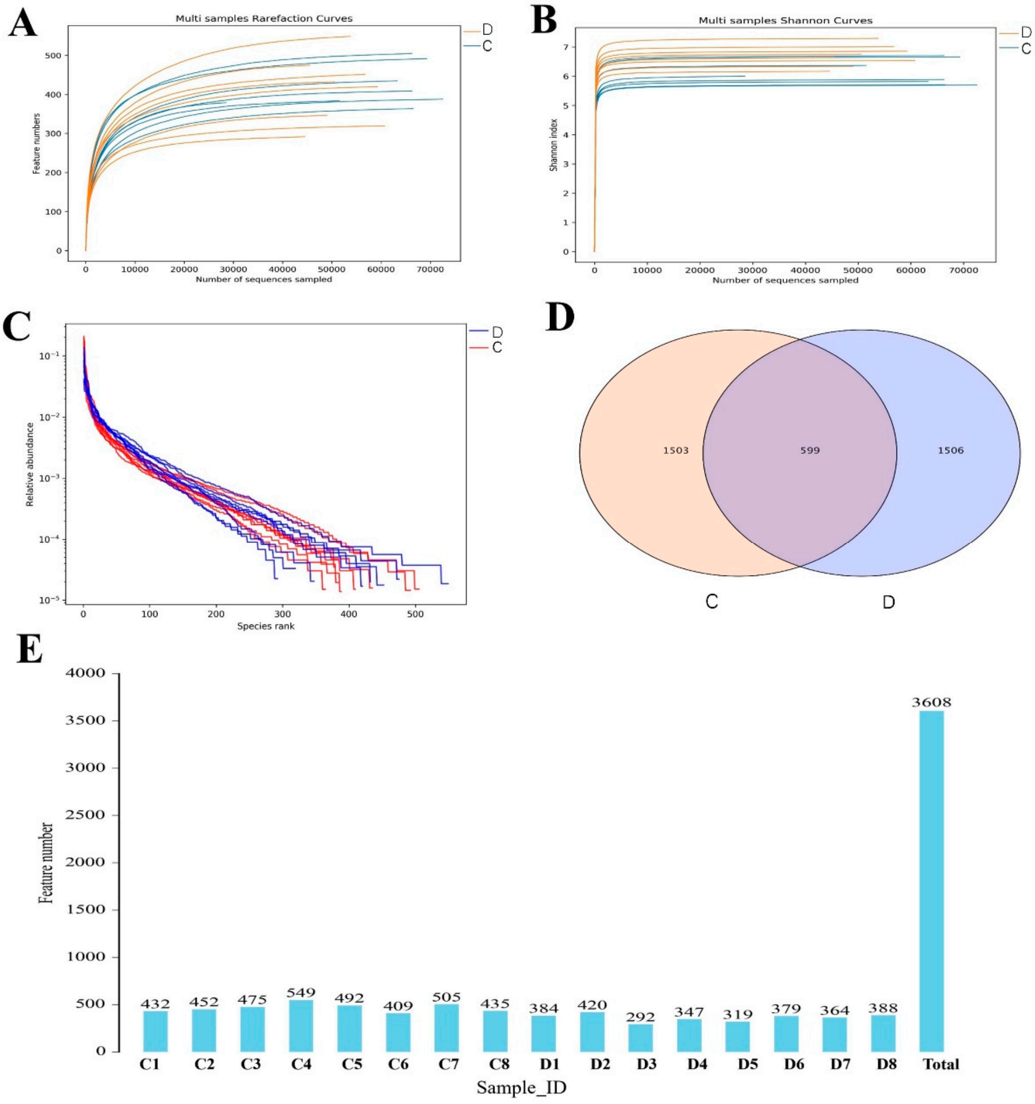

Figure S1. Analysis of sequencing depth and distribution of operational taxonomic units (OTUs) in the gut microbiota of groups C and D. The evaluation of gut microbial sequencing depth and evenness was performed using rarefaction (A, B) and rank (C) abundance curves. (D) Venn diagrams illustrate the distribution of common and distinct OTUs in each sample. (E）Quantity of OTUs in each sample.

Supplement Table S1

The information of the yak.

Sample	Age (60±5)	Gender	breed (Bos grunniens)	Body weight (kg)
C1 (Control Group1)	56	Male	maiwa yak	23.17
C2 (Control Group 2)	61	Male	maiwa yak	22.16
C3(Control Group 3)	60	Male	maiwa yak	23.63
C4(Control Group 4)	55	Male	maiwa yak	24.23
C5(Control Group 5)	62	Female	maiwa yak	21.36
C6(Control Group 6)	64	Female	maiwa yak	22.36
C7(Control Group 7)	65	Female	maiwa yak	24.03
C8(Control Group 8)	60	Female	maiwa yak	22.30
D1(Diarrhea Group1)	62	Male	maiwa yak	21.03
D2(Diarrhea Group2)	58	Male	maiwa yak	20.36
D3(Diarrhea Group3)	64	Male	maiwa yak	20.99
D4(Diarrhea Group4)	56	Male	maiwa yak	19.96
D5(Diarrhea Group5)	55	Female	maiwa yak	21.23
D6(Diarrhea Group6)	60	Female	maiwa yak	20.57
D7(Diarrhea Group7)	65	Female	maiwa yak	21.03
D8(Diarrhea Group8)	61	Female	maiwa yak	19.77

Table S2

Shifts in the gut microbial composition at the phylum and genus levels in yaks during diarrhea. C and D indicate control and diarrhea groups, respectively. Data were indicated as mean ± SD.

Taxa	C (%)	D(%)	P
**Bacteroidota**	11.54 ±1.64	51.25 ± 2.71	0.000999
**Firmicutes**	81.06 ± 1.32	35.53 ± 1.58	0.000999
**Proteobacteria**	0.51 ± 0.17	6.87 ± 1.63	0.00199
**unclassified_Lachnospiraceae**	31.60 ± 4.68	6.77 ±1.28	0.000999
**Lactobacillus**	14.65 ± 3.92	2.47±0.65	0.000999
**Lachnospiraceae_NK4A136_group**	8.90 ± 1.22	3.44± 1.30	0.013986
**unclassified_Muribaculaceae**	4.33 ± 0.63	22.95 ± 3.18	0.000999
**unclassified_Bacilli**	4.11 ± 1.19	1.08 ± 0.23	0.003996
**Alloprevotella**	1.18 ± 0.74	1.04 ± 3.29	0.013986
**Colidextribacter**	1.19 ± 0.29	0.26 ± 0.12	0.001998
**uncultured_Bacteroidales_bacterium**	0.98 ± 0.15	3.82 ±0.64	0.000999
**[Eubacterium]_xylanophilum_group**	0.79 ± 0.26	0. 05 ± 0.01	0.006993
**Lachnospiraceae_UCG_006**	0.45 ± 0.08	0. 07 ± 0.02	0.000999
**[Eubacterium]_siraeum_group**	0.43 ±0.18	0. 04 ± 0.02	0.003996
**Bacteroides**	0.43 ±0.11	5.60 ±1.67	0.000999
**unclassified_Peptococcaceae**	0.41 ± 0.08	0. 11 ±0.02	0.001998
**Rikenella**	0.22 ± 0.06	0. 55 ± 0.11	0.033966
**Parasutterella**	0. 22 ±0.13	4.92 ±1.59	0.002997
**Parabacteroides**	0. 18 ±0.13	1.29±0.42	0.001998
**Rikenellaceae_RC9_gut_group**	0. 09 ±0.02	0.49±0.07	0.000999
**uncultured_Muribaculaceae_bacterium**	0. 08 ± 0.01	0.26±0.04	0.001998
**UCG_005**	0. 08 ± 0.03	1.33±0.01	0.033966
**Muribaculum**	0. 07 ± 0.02	0.46±0.04	0.000999
**Dubosiella**	0. 06 ± 0.06	1.38±0.51	0.014985
**Family_XIII_UCG_001**	0. 05 ± 0.002	1.10±0.01	0.003996
**[Eubacterium]_ruminantium_group**	0. 04 ±0.04	0	0.000999
**unclassified_Cyanobacteriales**	0. 03 ±0.0009	0	0.001998
**Peptococcus**	0. 03 ±0.01	0	0.046953
**Escherichia_Shigella**	0.03 ±0.01	1.07±0.87	0.016983
**unclassified_Lactobacillaceae**	0. 02 ±0.007	0.00	0.010989
**Streptococcus**	0. 02 ±0.01	0. 99±0.35	0.003996
**uncultured_Clostridia_bacterium**	0. 02 ±0.01	0.00	0.027972
**unclassified_Clostridia**	0. 01 ±0.001	0. 07±0.02	0.018981
**[Acetivibrio]_ethanolgignens_group**	0. 01 ±0.001	0	0.000999
**UCG_003**	0. 01 ±0.0001	0	0.000999
**unclassified_UCG_010**	0. 01 ±0.0001	0.10±0.03	0.030969
**Pediococcus**	0. 01±0.0001	0	0.000999
**unclassified_Anaerovoracaceae**	0. 01±0.0001	0. 04±0.01	0.030969
**[Eubacterium]_oxidoreducens_group**	0. 01	0.00	0.016983
**Bacillus**	0.01	0.002	0.00099

**Limosilactobacillus**	0.01	0.00	0.00099
**[Eubacterium]_ventriosum_group**	**0.01**	**0.00**	**0.00099**
**Leuconostoc**	0.01	0.00	0.00099
**unclassified_Lactobacillales**	**0.01**	**0.00**	**0.00099**
**unclassified_Beijerinckiaceae**	0.01	0.00	0.00099
**Candidatus_Udaeobacter**	**0.01**	**0.00**	**0.00099**
**Dialister**	0.01	0.00	0.00099
**Fusobacterium**	**0.01**	**0.00**	**0.00099**
**Bifidobacterium**	0.00	0.50±0.17	0.008991
**Faecalibaculum**	0.00	0.18±0.08	0.003996
**Allobaculum**	0.00	0.79	0.000991
**Frisingicoccus**	0.00	0.09±0.08	0.000999
**Cetobacterium**	0.00	0.01±00	0.000999
**Coriobacteriaceae_UCG_002**	0.00	0.11±0.07	0.000999
